# Assessing the effect of sample storage time on viral detection using a rapid and cost-effective CTAB-based extraction method

**DOI:** 10.1186/s13007-024-01175-6

**Published:** 2024-05-08

**Authors:** Deogratius Mark, Fred Tairo, Joseph Ndunguru, Elisiana Kweka, Maliha Saggaf, Hilda Bachwenkizi, Evangelista Chiunga, James Leonard Lusana, Geofrey Sikazwe, Reuben Maghembe

**Affiliations:** 1 Tanzania Agricultural Research Institute, 6226, Dar-es-Salaam, Tanzania; 2 Tanzania Plant Health and Pesticides Authority, Arusha, P.O.Box 3024, Tanzania; 3 School of Aquatic Sciences and Fisheries Technology, University of Dar es Salaam, 60091, Dar es Salaam, Tanzania; 4 Mkwawa University College of Education, University of Dar es Salaam, 2513, Iringa, Tanzania; 5 Biological and Marine Sciences Unit, Faculty of Natural and Applied Sciences, Marian University College, 47, Bagamoyo, Tanzania; 6 Department of Biological Sciences, Faculty of Science, University of Botswana, Private Bag 0704, Gaborone, Botswana

**Keywords:** CTAB-method, DNA/RNA quality, DNA/RNA degradation, Storage time

## Abstract

**Background:**

Cassava leaf samples degrade quickly during storage and transportation from distant areas. Proper sampling and efficient, low-cost storage methods are critical for obtaining sufficient quality DNA and RNA for plant virus epidemiology and improving disease control understanding. This is useful when samples are collected from remote areas far from a laboratory or in developing countries where money and materials for virus diagnostics are scarce.

**Results:**

The effect of sample storage duration on nucleic acid (N.A.) quality on virus detection was investigated in this study. A simple, rapid, and cost-effective CTAB-based approach (M3) for single N.A. extraction was optimized and tested alongside two existing CTAB-based methods (M1 and M2) for N.A. extraction from fresh and herbarium cassava leaves stored for; 1, 8, 26, and 56 months. The amount and quality of DNA and RNA were determined using Nanodrop 2000 c U.V.–vis Spectrophotometer and agarose gel electrophoreses. The sample degradation rate was estimated using a simple mathematical model in Matlab computational software. The results show no significant difference in mean DNA concentration between M1 and M2 but a significant difference between M3 and the other two methods at *p* < 0*.*005. The mean DNA concentration extracted using M3 was higher for 1 and 8 months of leave storage. M3 and M2 produced high concentrations at 26 and 56 months of leave storage. Using a developed scale for quality score, M3 and M2 produced high-quality DNA from fresh samples. All methods produced poor-quality DNA and RNA at 8 and 26 months of leave storage and no visual bands at the age of 56 months. Statistically, there was a significant difference in the mean DNA quality between M1 and M2, but there was no significant difference between M3 and the other two methods at *p* < 0*.*005. However, Cassava brown streak virus (CBSV) and Ugandan cassava brown streak virus (UCBSV) were readily detected by RT-PCR from RNA isolated using M3. The quality of DNA declined per storage time at 0.0493 and 0.0521/month, while RNA was 0.0678 and 0.0744/month. Compared to the existing two methods, modified CTAB extracted enough high-quality N.A. in one-third the time of the existing two methods.

**Conclusion:**

Our method provides cost-effective, quick, and simple processing of fresh and dry samples, which will quicken and guide the decision on when and what type of sample to process for plant disease management and surveillance actions.

**Supplementary Information:**

The online version contains supplementary material available at 10.1186/s13007-024-01175-6.

## Background

Although collecting plant samples in liquid nitrogen and storing them at – 80 °C is the most effective storage method for most plant species; it is not feasible in many developing countries due to high costs and difficulties in obtaining the necessary resources. Providing continuous power supplies for ultralow (− 80 °C) freezers may be complicated and costly [1]. Thus, chemical treatments of samples with formalin or ethanol have been commonly used as collection and storage methods in developing countries, even though both cause significant DNA degradation [2, 3]. Herbarium also provides a chemical-free and low-cost alternative to sample storage and has been used for taxonomical studies, with researchers able to recover ancient DNA from plant and animal samples dating back thousands of years [4–6], contrary to popular belief,

‘time’ is a demonized factor in sampling genetic material, causing severe degradation. However, proper drying methods for herbarium plant tissue preservation are required because improper and rapid drying procedures, such as using artificial heat, minimize extreme water stresses, shortage of nutrients, and wounding of tissues, which induces the production of phenolic compounds and free radicals, which can interfere with DNA extraction and/or amplification [7]. Therefore, proper handling of plant tissue samples and storage to maximize shelf life is crucial in molecular and diagnostic studies.

The cetyltrimethylammonium bromide (CTAB) method of nucleic acid extraction has become a reliable method for many applications in plant science in recent years. Murray and Thompson [8] made a momentous discovery of the CTAB method nearly four decades ago, contributing to our understanding of what constitutes a ‘cost-effective’ method for nucleic acid isolation. The technique was significant because it could isolate DNA and RNA from different plant species [8], and it was reported to be cheaper than column-based technology [9] and guanidium thiocyanate-phenol extraction [10]. Significant effort has been expended in the search for a method that is sensitive, reproducible, cost-effective, time-efficient, and yields high-quality DNA and/or RNA for PCR-based detection of plant pathogens such as *cassava brown streak viruses* and *cassava mosaic begomoviruses* [9], begomoviruses from jute and other mucilaginous crops [11], diverse plant pathogens (RNA and DNA viruses, viroids, phytoplasmas, and bacteria) that infects plants host such as sweet potato, small fruits, and fruit trees [12], viroids, DNA and RNA viruses in tomato, potato, and citrus [13].

For many years, the CTAB method has been the preferred method of extracting nucleic acids from fresh cassava leaf samples for many molecular studies [9, 14]. It is still the most reliable and cost-effective method of extracting nucleic acids, ten times cheaper than commercial kits [9]. Even though post-sampling DNA degradation occurs in herbarium-stored samples and is known to increase post-sampling, it has been proposed that DNA sequencing using NGS obtained from fresh samples and stored samples is reliable in different scientific investigations [15].

We tested this assumption by comparing the yield of genetic materials from fresh cassava and herbarium samples of varying ages. Our main focus was to evaluate the impact of sample storage time on the quality of DNA and RNA. To achieve this, we made modifications to the CTAB-based approach for nucleic acid isolation and compared it with two standard CTAB methods. Our goal was to develop a time-efficient and cost-effective extraction method by reducing the required time, reagents, and consumables. We isolated DNA and RNA from fresh and herbarium cassava leaf samples that had been stored for different durations: 1, 8, 26, and 56 months. These samples were then compared in terms of their quality, quantity, and purity. Our optimized protocol proved to be swift, taking less than 29 min, while the two ordinary methods required 95 and 110 min, respectively. Interestingly, using our optimized method, we were able to detect the presence of the Cassava brown streak virus (CBSV) and Ugandan cassava brown streak virus (UCBSV), both of which are RNA viruses belonging to the genus Ipomovirus, even in degraded RNA samples. This finding challenges the common belief that degraded RNA is unsuitable for virus detection. It is worth noting that the degraded samples had been stored for over 5 years. Moreover, for the first time, we developed a quality score for nucleic acids based on the band intensity of the optimal amount obtained through agarose gel electrophoresis. This study emphasizes the importance of using known quantities during quality control validation of nucleic acids to avoid erroneous judgments. Additionally, we used a mathematical model to predict the rate of RNA and DNA degradation in cassava genetic materials stored for different durations. These predictions can be instrumental in addressing common delays in procurements, limited availability of materials and resources, and overall streamlining laboratory operations and management. Furthermore, our findings can guide the development of efficient protocols for DNA and/or RNA isolation, as well as diagnostic methods for virus detection and identification in the developing world.

## Methods

### Sample collection and storage

Six herbaria-pressed cassava brown streak disease (CBSD)-symptomatic cassava leaf samples were collected in five age groups from different districts in Tanzania between 2015 and 2019. The samples were 56 month-old of the variety Kalingisi, 26 month-old of the variety Mkombozi, and 8- and 1 month-old of an unknown variety. Fresh samples of unknown variety were collected in 2019 from a farmer’s field in Kimara in Dar Es Salaam and stored at 4 °C for laboratory analysis on the same day.

### Isolation of genomic DNA and total RNA

Three CTAB-based extraction methods referred to in this paper as Method 1 (M1), Method 2 (M2), and Method 3 (M3) were used in this study. M3 was an optimized protocol of M1 [9] and M2 which is commonly used in our laboratory optimized from Lodhi [16]. M1 and M2 were performed exactly as described in the original protocols.

#### Method 1

**Fig. 1:**
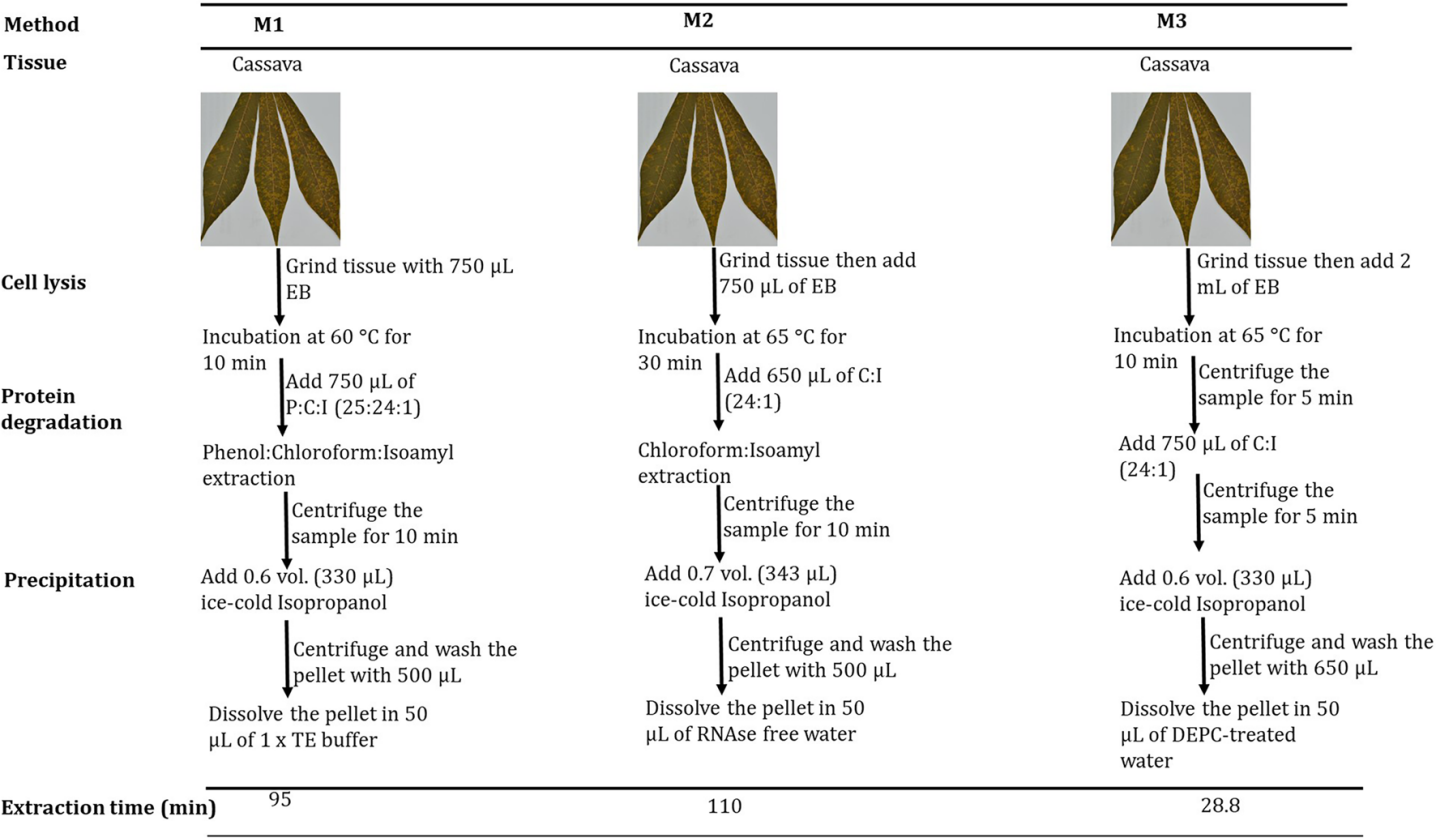
Schematic diagram showing different extraction steps used by all three methods from initial (mechanical step) to the recovery step. EB; extraction buffer, P:C:I; Phenol: Chloroform: Isoamyl alcohol

This method was modified from [16, 17] by [9]. About 100 mg of fresh and dried leaf tissue were ground using mortar and pestles. After adding about 1 mL of CTAB buffer to the ground leaf tissue, 750 µL was transferred to a sterile 1.5 mL Eppendorf tube. The homogenate was thoroughly mixed and incubated at 60 °C for 10 min. The extract was then mixed with equal volume (750 µL) of phenol: chloroform: isoamyl alcohol (25:24:1) and centrifuged at 13,000 × g (> 12,000 × g) for 10 min. The supernatant was transferred to a new sterile Eppendorf tube, and total nucleic acids were precipitated by adding 0.6 volume of ice-cold isopropanol (− 20 °C). After incubating at − 20 °C for 60 min, samples were centrifuged at 12,000 × g for 10 min at + 4 °C, and the pellet was washed in 500 µL of 70% ethanol before being centrifuged at 12,000×*g* for 5 min and air-dried at room temperature. The pellet was dissolved in 50 µL 1 × T.E. buffer and stored at – 20 °C for further analysis. The summary of the extraction process for M1, M2, and, M3 is summarized in Fig. 1.

#### Method 2

This method was modified by the cassava diagnostic project team of Agricultural Research Institute-Mikocheni (now Tanzania Agricultural Research Institute) from formerly described protocols by [16, 18]. Using sterile mortars and pestles, 150 mg of fresh and dried cassava leaf sample was ground before adding an extraction buffer. About 750 µL of CTAB buffer warmed at 65 °C was added to the ground samples, and 650 µL of homogenate was transferred to a 1.5 mL Eppendorf tube and then vortexed to dispense the tissue in the buffer. The samples were incubated at 65 °C for 30 min, mixed by inversion every 10 min, and left at room temperature for 10 min to cool. An equal volume (650 µL) of chloroform: isoamyl alcohol (24:1) was added to the samples, mixed by inversion for 10 min, and centrifuged at 12,000 rpm for 10 min. The supernatant (about 500 µL) was transferred to a sterile Eppendorf tube, and 0.7 volume (343 µL) of cold (− 20 °C) isopropanol was added and incubated at – 20 °C for 30 min, then centrifuged at 13,000 rpm for 10 min. The pellet was washed by adding 500 µL of 70% ethanol and spun at 13,000 rpm for 10 min. The samples were air-dried for 40 min and re-suspended in 50 µL RNAse-free water.

#### Method 3

The optimized method described in this study included minor modifications (Table 1); 100 and 50 mg of fresh and dried leaf tissue, respectively, were ground using a sterile mortar and pestle. About 2 mL of extraction buffer (2% CTAB, 2% PVP40,000, 25 mM EDTA pH 8, 100 mM Tris–HCl pH 8, 2.5 mM NaCl) pre-warmed at 65 °C was added to the ground leaf samples and incubated at 65 °C for 10 min. The homogenate was centrifuged at 16,708 ×*g* for 5 min at 4 °C, and the supernatant (750 µL) was transferred to new sterile 1.5 mL Eppendorf tubes. An equal volume (750 µL) of chloroform isoamyl alcohol (24:1) was added and centrifuged at 16,708 ×*g* for 5 min. The supernatant (550 µL) was transferred to new 1.5 mL Eppendorf tubes, and 0.6 volumes of cold isopropanol were added and centrifuged again at 16,708 ×g for 5 min to form pellets. Isopropanol was decanted, and about 650 µL of 70% DEPC-treated ethanol was added onto the pellets, centrifuged at 17,968 ×*g* for 3 min, and air-dried before re-suspending into 40 µL of DEPC-treated water. The nucleic acid quality and integrity were checked by a 1.0% agarose gel electrophoresis stained with 0.1 mg/mL ethidium bromide in 100 mL of 1 × TAE buffer solution. The gel was viewed under U.V. light using a gel documentation machine (BioDoc-It 210 Imaging Systems, Upland, CA, USA).

**Table 1:** The final concentration of reagents used in a single extraction of genomic DNA and total RNA. M1, M2 and, M3 are methods 1, 2 and, 3, respectively

Extraction methods (M)		M1	M2	M3
Parameter	Reagent/buffers			
Reagents	CTAB powder NaCl EDTA pH Tris–HCl pH 2-mercaptoethanol	2% 1.4 M 20 mM 100 mM 0.2%	2% 1.4 M 20 mM 100 mM 5%	2% 2 M 25 mM 100 mM N/A
	PVP	N/A	N/A	2%
Extraction step (Time in minutes)	Cells lysis^a^ Lipid-debris partitioning^b^	10 10	30 20	10 5.8
	Nucleic acids recovery^c^	60	30	N/A
	Other steps^d^	15	30	13
	Total extraction time	95	110	28.08

Where (^a^) represents CTAB buffer/homogenate incubation time, (^b^) is chloroform: isoamyl/phenol:chloroform: isoamyl alcohol inversion time and centrifugation, (^c^) cold isopropanol/precipitation incubation time and (^d^) represents isopropanol/ethanol and prior adding chloroform: isoamyl centrifugation time

### Quality check validation

Nanodrop 2000c U.V.–vis Spectrophotometer (Thermo Scientific, Wilmington, DE, USA) was used to take the spectrophotometric readings of DNA to determine its concentration (ng/µL) and purity at 260/280 and 260/230 absorbance ratios. Prior separation of genomic DNA and total RNA in the agarose gel, the total nucleic acids extracted from fresh leaf samples using M3 were normalized to obtain ten amounts of 0.2 ng, 0.4 ng, 0.6 g, 0.8 ng, 1.0 ng, 1.2 ng, 1.4 ng, 1.6 ng, 1.8 ng, and 2.0 ng. These amounts were loaded in the gels to determine the smallest amount that produced a clear, bright band and used as standard in subsequent gels. The DNA and RNA were stained with ethidium bromide, separated by 1.5% agarose gel electrophoresis according to their molecular size, and visualized in the gel documentation machine.

### Development of DNA and RNA quality score

Based on gel pictures obtained after running a standard amount, a scale of 0 to 3 was developed based on the intensity of the nucleic acid band, with 0 representing degraded N.A. (no band), 1 representing poor quality (very faint) DNA/RNA bands, 2 representing moderate (clear band but not bright) and 3 representing high-quality bands.

### Synthesis of complementary DNA (cDNA)

The RNA templates were reversed transcribed into complementary DNA (cDNA) using 1 µL (200 U/µL) of Moloney Murine Leukemia Virus Reverse Transcriptase (M-MuLV RT; M0253; New England Biolabs (NEB); Ipswich, Massachusetts, USA) using the quick protocol. Other R.T. components added to the reaction, including 1 µL of 100 M random hexamer (Bioneer, Seoul, South Korea), 1 µL of 10 mM dNTPs (NEB), 2 l of 10X M-MuLV buffer, 0.2 l of 40 U/µL of RNase inhibitor, 5 µL of 200 ng/µL of total RNA and DEPC water to a volume of 20 µL. The R.T. reaction was extended at 42 °C for 1 h, and the enzyme was inactivated at 65 °C for 20 min.

### PCR-based detection of *Cassava brown streak virus* (CBSV) and *Ugandan cassava brown streak virus* (UCBSV)

The CBSV and UCBSV were detected using the primer pair for simultaneous detection of both viruses CBSDDF2 (GCTMGAAATGCYGGRTAYACAA) and CBSDDR (GGATATGGAGGAAGRKCTCC) [19], which amplifies the part of coat protein and HAM1 gene with the expected size of 344 base pairs for CBSV and 440 base pairs for UCBSV. The amplifications were in 25 µL final reaction volumes containing 0.5 µL (0.2 µM) of each primer, 12.5 µL (1X) of OneTaq Quick-Load, 2X Master with Standard Buffer (New England Biolabs Inc.), 10.5 µL Nuclease-free water and 5 µL of diluted cDNA template. PCR amplification was carried out in a thermal cycler programmed for initial denaturation at 94 °C for 30 s, followed by 30 cycles of denaturation at 94 °C for 30 s, annealing temperature was at 51 °C for 45 s, and extension at 68 °C for 1 min. The last extension step was accomplished at 68 °C for 5 min at the end of the amplification reaction. The chloroplast ribulose-bisphosphate carboxylase gene (rbcL) of 599 bp, the barcode DNA for plants, was amplified from fresh samples of all age categories using P609-GTAAAATCAAGTCCACCRCG and P610-ATGTCACCACAAACAGAGACTAAAGC primer pairs [20] similar to PCR cocktail as described above. The PCR was performed in an Applied Biosystems GeneAmp PCR System with a heated lid under the following conditions: an initial denaturation (95 °C, 30 s) followed by 30 cycles with a denaturation of 94 °C for 30 s, an annealing of 57 °C for 45 s, an extension of 68 °C for 1 min, and a final extension of 68 °C for 5 min. The PCR products were run on 2% agarose gels stained with 0.1 mg/mL ethidium bromide for 1 h at 180 V. Gel images were captured using a Benchtop U.V. Transilluminator (UVP; Upland, CA, USA) under U.V. light.

### Estimation of RNA and DNA degradation rate

A simple mathematical model was formulated based on chemical reactions to predict nucleic acid degradation rates. Briefly, we define *Q* as the quality of RNA/DNA over time, and *k* is the degradation coefficient per unit of time. The rate of change in RNA/DNA quality is governed by the following (eq. 1).

1 $$\frac{dQ}{{dt}} = - kQ;\,\,\,\,\,Q\left( 0 \right) = Q_{0}$$

whereby *Q*_0_ is the quality of RNA/DNA for freshly isolated leaf samples. The solution to (i) is an exponential decay function given by

2 $$Q\left( t \right) = \,Q_{0} e^{ - kt}$$

An exponential function is appropriate for modeling data that either increases or decreases over time. It has been used elsewhere to estimate the degradation of environmental DNA and RNA in a marine system [21]. We used eq. 2, together with laboratory data obtained in this study, to estimate the rate of sample degradation. To determine the rate at which RNA/DNA degrades, we match equation ii with the score data (Table 2) so that the difference between them is as minimum as possible.

**Table 2:** DNA and RNA quality scores for different sample storage periods and extraction methods.

NA	ID	Sample periods														
		Fresh			1 Month			8 Months			26 Months			56 Months		
		M1	M2	M3	M1	M2	M3	M1	M2	M3	M1	M2	M3	M1	M2	M3
DNA	1	3	2	3	2	2	1	1	1	1	1	1	1	0	0	0
	2	2	3	3	1	2	2	1	1	1	1	1	1	0	0	0
	3	2	3	3	0	2	2	1	1	1	1	1	1	0	0	0
	4	3	3	3	0	2	1	1	1	1	1	1	1	0	0	0
	5	3	3	3	1	2	2	1	1	1	1	1	1	0	0	0
	6	2	3	3	0	2	1	1	1	1	1	1	1	0	0	0
RNA	1	1	2	3	3	2	3	1	1	2	1	0	1	0	0	0
	2	1	2	3	1	2	3	1	1	2	0	0	1	0	0	0
	3	3	2	3	2	2	3	1	1	2	0	0	1	0	0	0
	4	3	2	3	2	2	3	1	1	2	1	0	1	0	0	0
	5	3	2	3	3	2	3	1	1	2	1	0	1	0	0	0
	6	3	2	3	3	2	3	1	1	2	1	0	1	0	0	0

Scores designed in this study follow a 0 to 3 scale, where degraded N.A. is denoted by 0, 1 for poor-quality DNA/RNA bands, 2 for moderate, and 3 for high-quality bands. M1, M2, and M3 represent method 1, method 2, and optimized method 3 of N.A. extraction

### Data analysis

Data collected on the quantity and quality of DNA and RNA in samples with different storage times and N.A. extraction methods were compared using R software and presented as box plots. The quality of DNA and RNA was assessed using a score scale explicitly developed for this study, with a scale of 0 representing samples with no visible band on gel electrophoresis, a score of 1 representing low-quality band intensity, a score of 2 representing moderate band quality, and a score of 3 representing high band quality. A simple mathematical model in the Matlab (9.11.0.1809720 (R2021b) computational software was used to determine the rate at which leaf tissues deteriorate and produce low-quality nucleic acids. Two datasets were created to ensure the robustness of the method used to estimate RNA/DNA degradation rate: (1) by averaging RNA/DNA quality obtained by three different isolation methods and (2) by averaging RNA/DNA quality for six leaf samples used in each extraction method. Analysis of variance was used to test for differences between the concentration of DNA and DNA and RNA quality. The means of DNA concentration and DNA and RNA quality were separated by Fisher’s protected test at least significant difference using the GenStat 15th edition.

## Results

### DNA and RNA quantity and quality based on sample storage periods

**Fig. 2:**
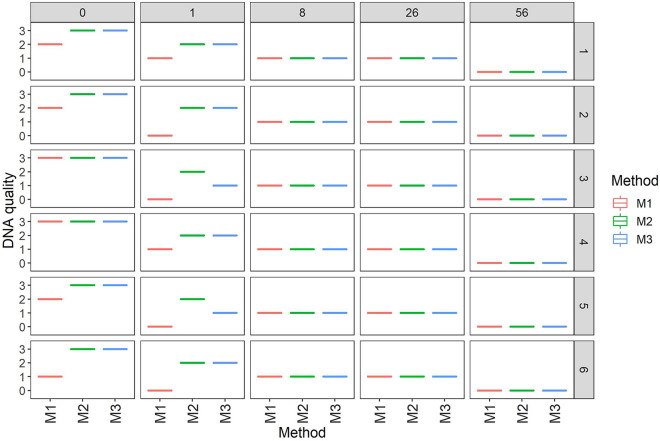
Box plots illustrating the quality of DNA using generated scale of 0 to 3. The single line indicates either of four scales (0–3) which represent a single band per sample. A scale of 0 to 3 is based on visual observation of DNA bands in the gels where 0-no band, 1-low quality (poor), 2-moderate quality, and 3-high quality. The right y-axis represents six different varieties where the samples were collected (details in subsection one of the methods section)

**Fig. 3:**
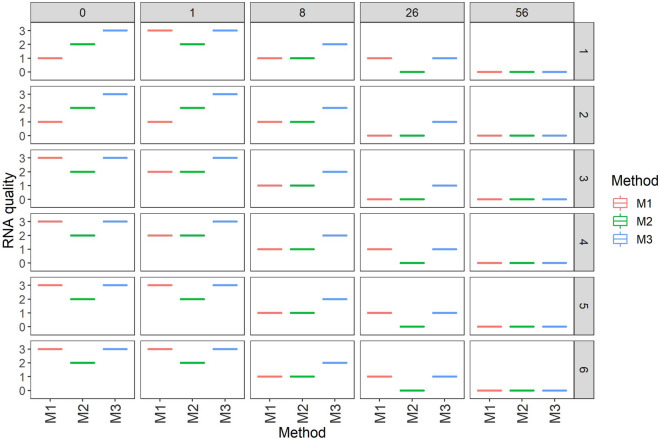
Box plots illustrating the quality of RNA using a generated scale of 0 to 3. The single line indicates either of four scales (0–3) which represent a single band per sample. A scale of 0 to 3 is based on visual observation of RNA bands in the gels where 0-no band, 1-low quality (poor), 2-moderate quality, and 3-high quality. The right y-axis represents six different varieties where the samples were collected (details in subsection one of the methods section)

The quality of DNA and RNA was found to decrease as sample storage time increased. All extraction methods yielded significant DNA and RNA from fresh leaf samples, but they were of poor quality in 8- and 26 month-old herbarium samples. In 56-month-old herbarium-stored samples, no bands were observed (Figs. 2, 3), implying that nucleic acid quality decrease as sample storage time increases. It was observed that cassava leaves stored for a more extended period had significantly lower DNA and RNA quality than those stored for a shorter period (*p* < 0*.*005, Table 3). In all extraction methods, the quality of DNA and RNA extracted from tissues stored for one day and one month was significantly higher than that obtained from tissues stored for 8, 26, and 56 months. The quality of DNA and RNA did not vary considerably for 8, 26, and 56 months between extraction methods (Table 2).

**Table 3:** The Concentration of DNA, quality of DNA, and RNA at different storage times and methods

Time/Methods	^a^Conc. (ng/µL)	^a^QDNA	^a^QRNA
T0M1	1473ab	2.167e	2.333c
T0M2	824a	3f	2c
T0M3	1257a	3f	3d
T1M1	701a	0.333b	2.333c
T1M2	794a	2e	2c
T1M3	3050c	1.667d	3d
T8M1	1109a	1c	1b
T8M2	758a	1c	1b
T8M3	2749c	1c	2c
T26M1	1323a	1c	0.667b
T26M2	1475ab	1c	0a
T26M3	2286bc	1c	1b
T56M1	1253a	0a	0a
T56M2	2424c	0a	0a
T56M3	2340bc	0a	0a
Fpr	< 0*.*001	< 0*.*001	< 0*.*001
LSD 5%	913.7	0.6038	0.4200
CV%	50.0	23.1	26.9

T0 (time 0) represents the fresh sample results obtained using M1, M2, and M3. T1, T8, T26 and, T56 represent the storage times 1, 8, 26 and, 56 months, respectively. QDNA and QRNA are the quality of DNA and RNA, respectively

^a^Mean concentration and mean quality of nucleic acids. Different letters within each column indicate significant differences (P < 0.05). For all variables with the same letter, the difference between the mean concentration and mean quality is not statistically significant (p < 0.05) [43–45]

### DNA and RNA quantity and quality based on the extraction method

**Fig. 4:**
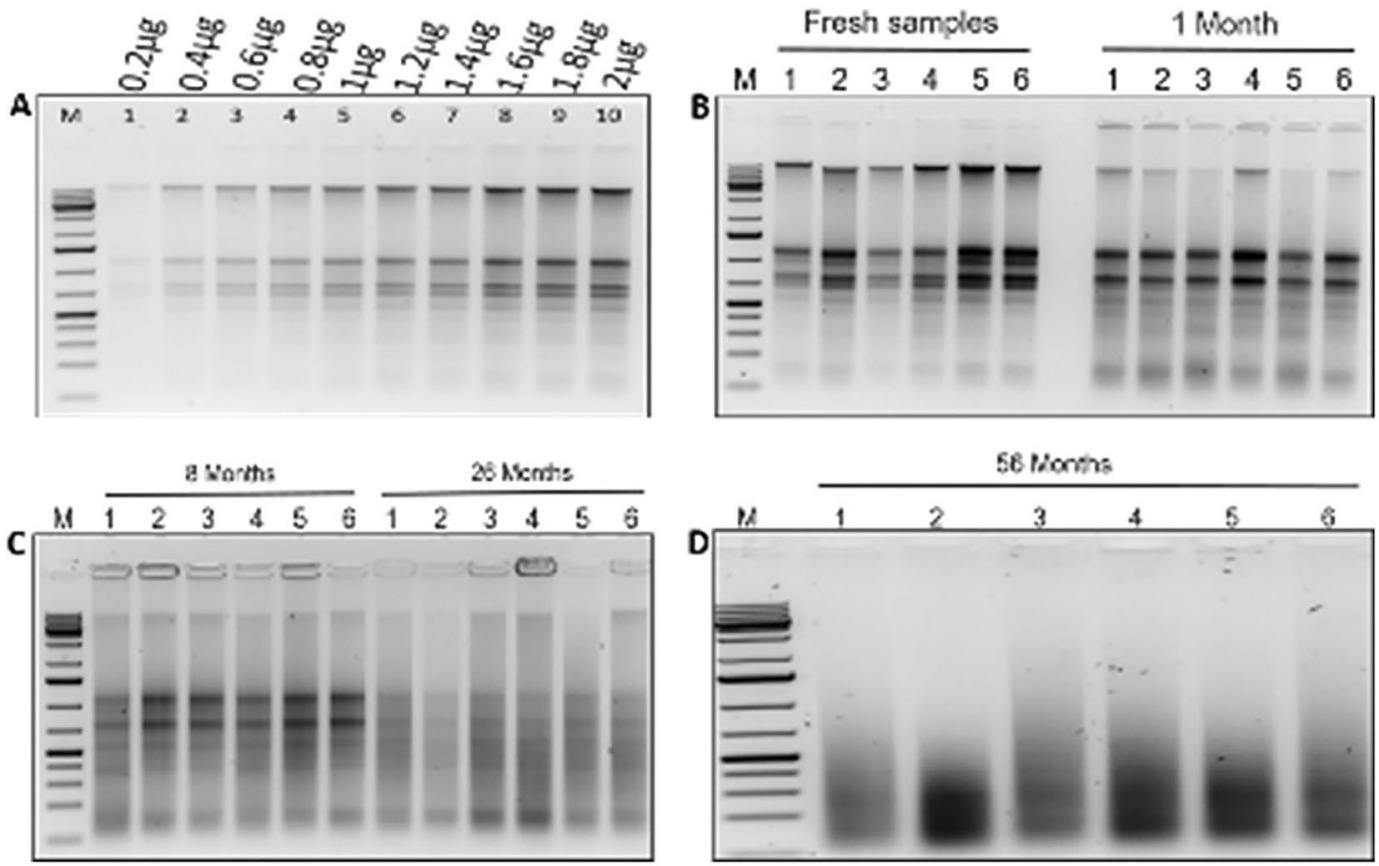
Total nucleic acids isolated using the modified method (M3). Gel picture **A **= ten different dilutions used to obtain the optimal amount of nucleic acid to use for the samples in the study; Gel picture **B** = nucleic acids from fresh and 1 month samples; Gel picture ** C** = 8- and 26 month old samples; Gel picture D = 56 months old samples showing smears and no evidence of DNA and RNA

**Fig. 5:**
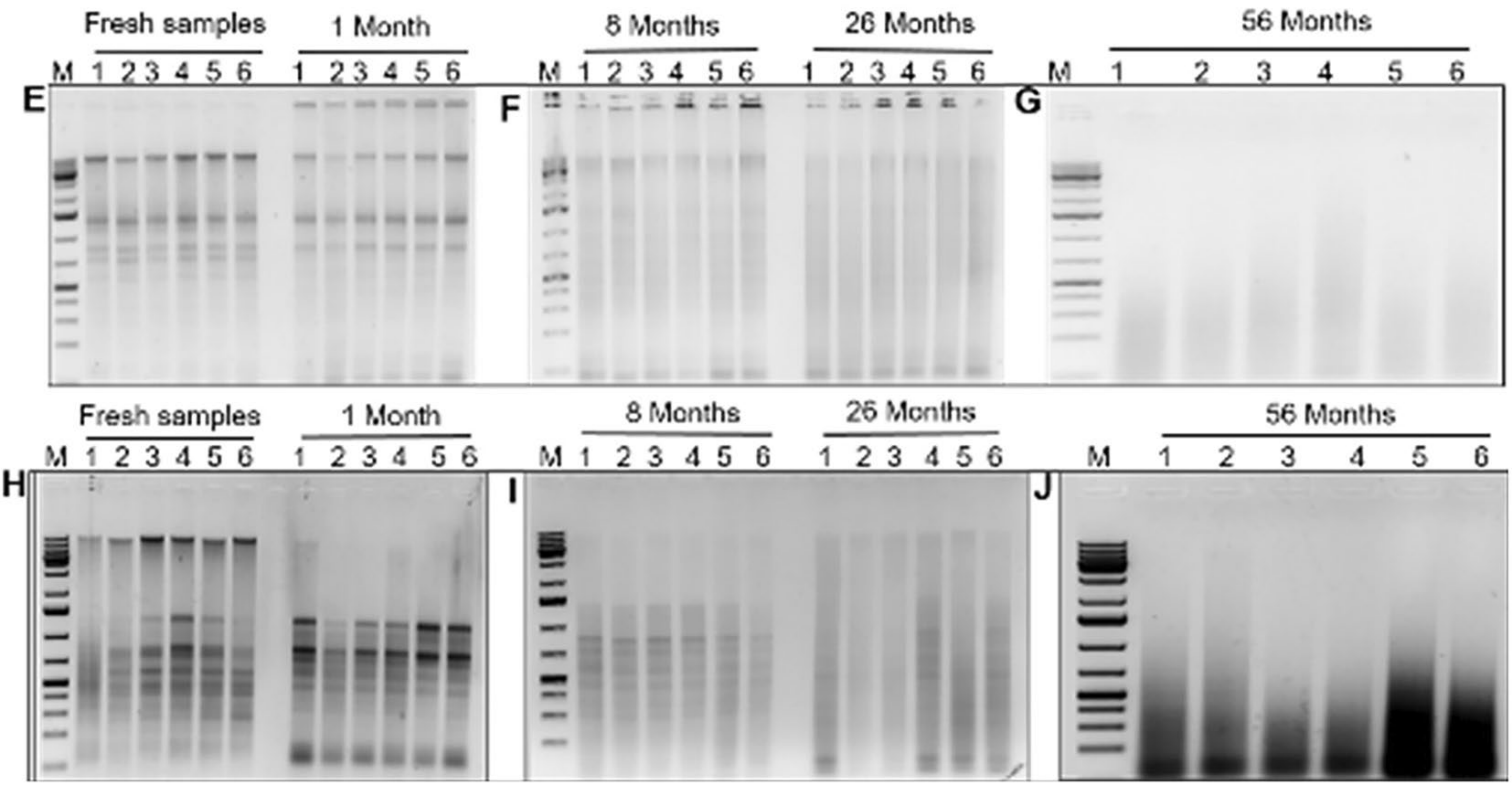
Total nucleic acids isolated using extraction methods M1 and M2. Gel pictures **E**, **F** and **G**, show bands of nucleic acids from fresh samples and those aged 1, 8, 26, and 56 months using extraction M2. Gel pictures **H**, **I** and **J** shows nucleic acids of fresh samples, and those aged 1, 8, 26, and 56 months using extraction M1

The amount and quality of DNA and RNA obtained using the existing CTAB methods, M1 and M2 were generally low compared to M3 used in this study. Except for the 56 month-old herbarium-stored samples, our modified method recovered sufficient high-quality DNA and RNA than the existing two CTAB methods (Fig. 4, 5). M2 and M3 recovered high-quality DNA from all fresh leaf samples, whereas M1 recovered moderate-quality DNA. Again, M2 and M3 generated moderate-quality DNA from 1-month-old samples, whereas M1 produced poor-quality DNA. For 56 month-old samples, all methods failed to recover DNA (Fig. 4, Tables 2, 4). The results show no significant difference in mean DNA concentration between M1 and M2, but there is a significant difference between M3 and the other two methods at p < 0.005. The mean concentration of DNA extracted with M3 was higher at 1 and 8 months, and M3 and M2 produced high concentrations at 26 and 56 months old, respectively (Tables 2, 4).

**Table 4:** Concentration of DNA in ng/µL, quality of DNA and RNA at different storage times (0: Fresh leaves, 1: One month, 8: 8 months, 26: 26 months, and 56: Fifty-six) and methods of extraction used (M1, M2, and M3)

Sample ID	Time (M)	Concentration ng/µL			Quality of DNA			Quality of RNA		
		M1	M2	M3	M1	M2	M3	M1	M2	M3
1	0	2236.4	963.7	1514.2	2	3	3	1	2	3
2	0	1394.6	630.7	1411.1	2	3	3	1	2	3
3	0	562.9	630.9	1546.9	3	3	3	3	2	3
4	0	1415.6	795.4	852.1	3	3	3	3	2	3
5	0	1097	1014.5	595.5	2	3	3	3	2	3
6	0	2133.7	910.9	1623.3	1	3	3	3	2	3
1	1	1257	913.6	2301.5	1	2	2	3	2	3
2	1	598	795.9	2763.7	0	2	2	1	2	3
3	1	1136.2	693	4815.6	0	2	1	2	2	3
4	1	531	801.3	1766.2	1	2	2	2	2	3
5	1	391.8	589.9	2375.6	0	2	1	3	2	3
6	1	290.6	973.3	4274.4	0	2	2	3	2	3
1	8	799.3	699.9	5744.3	1	1	1	1	1	2
2	8	574.8	571.6	3570.7	1	1	1	1	1	2
3	8	1200.8	707.8	2236.4	1	1	1	1	1	2
4	8	500.2	575.1	1393.4	1	1	1	1	1	2
5	8	2868.8	572.9	1415.6	1	1	1	1	1	2
6	8	710.1	1418.3	2133.7	1	1	1	1	1	2
1	26	2354	1440.1	2031.3	1	1	1	1	0	1
2	26	900.5	1351.7	1614	1	1	1	0	0	1
3	26	656.8	425.2	2868.8	1	1	1	0	0	1
4	26	531.6	2310.8	2354	1	1	1	1	0	1
5	26	2400.2	1254.9	1390	1	1	1	1	0	1
6	26	1093	2070.1	3455.3	1	1	1	1	0	1
1	56	826.8	2987.6	4125.3	0	0	0	0	0	0
2	56	1411.3	3135.1	1449.6	0	0	0	0	0	0
3	56	1896.2	2115.5	2362.7	0	0	0	0	0	0
4	56	785.1	2175.5	2990	0	0	0	0	0	0
5	56	1227	2764.1	1758.7	0	0	0	0	0	0
6	56	1373.7	1367.3	1351.6	0	0	0	0	0	0

In terms of RNA yield using M3, fresh and 1 month-old samples produced high-quality DNA, while M2 produced moderate-quality RNA. M3 generated moderate-quality RNA on 8 month-old herbarium-stored samples, whereas M1 and M2 detected low-quality RNA on the same samples. M3 and M1 recovered low-quality RNA from 26-month-old samples, while M2 produced no visible RNA bands. None of the three methods recovered RNA from 56-month-old herbarium samples (Tables 2, 4). We reduced DNA and RNA extraction time in M3, which extracted enough total nucleic acids of high quality in one-third of the time (28/95 min) that the other two methods take (Table 5).

**Table 5:** Extraction steps and time taken by each method to extract N.A. from incubation step to recovery

Extraction steps	Time (min)	M2	M3
	M1		
Incubation at 60–65 °C	10	40	10
Mixing with C: I or P:C: I by inversion	Not indicated	10	0.08
Centrifugation (total)	25	30	18
Incubation at − 20 °C	60	30	0
Total extraction	95	110	28.08

### RNA and DNA degradation rate

**Fig. 6:**
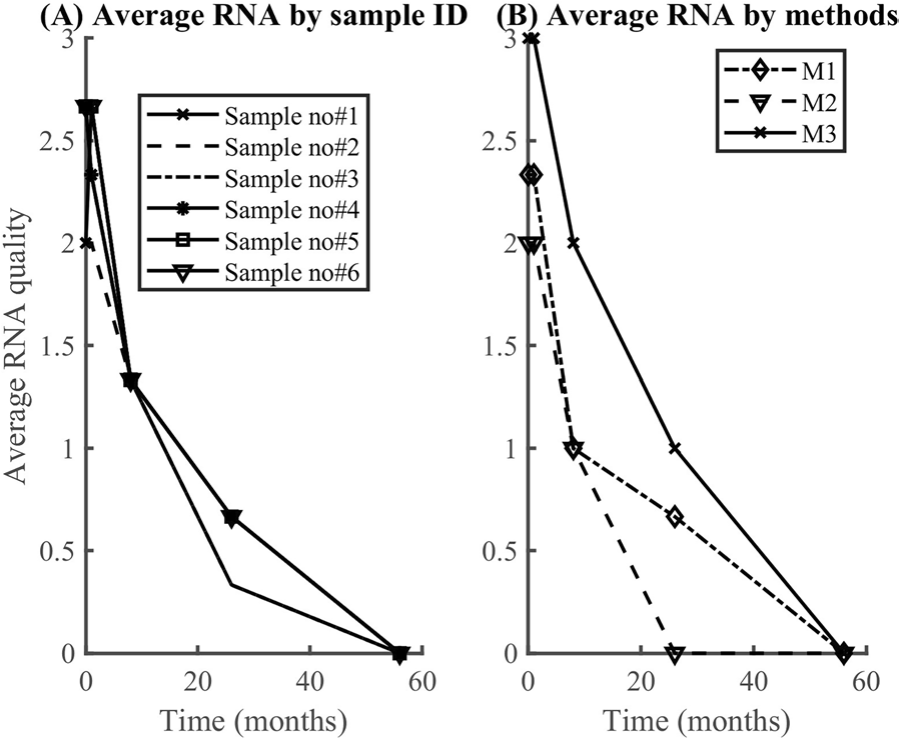
RNA quality score data aggregated by **A** six cassava leaf samples and **B** three CTAB- based extraction methods

**Fig. 7:**
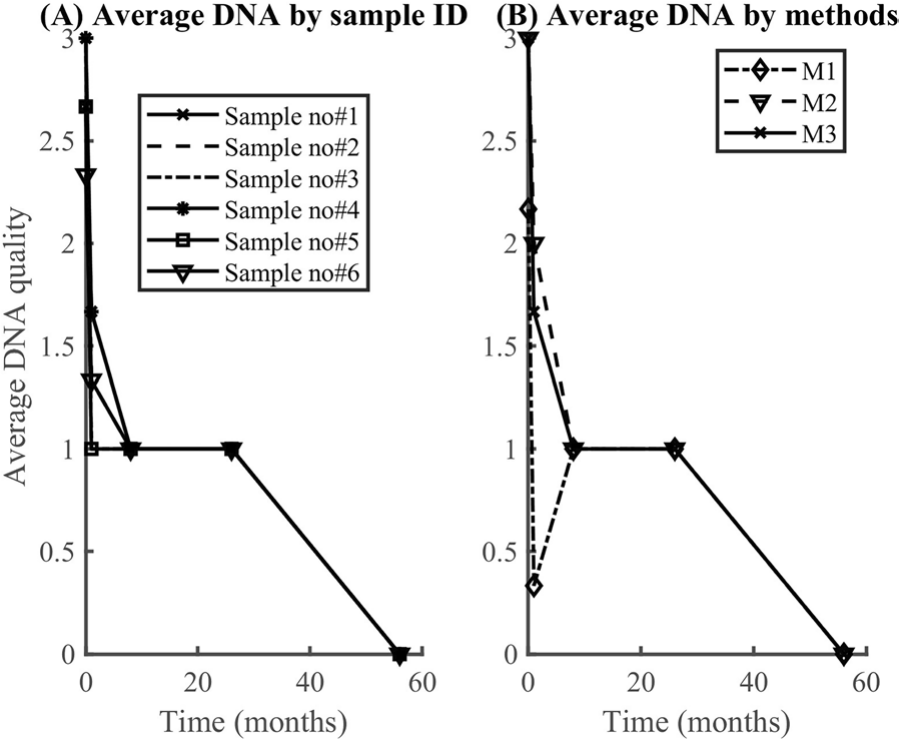
DNA quality score data aggregated by **A** six cassava leaf samples and **B** three CTAB- based extraction methods

**Fig. 8:**
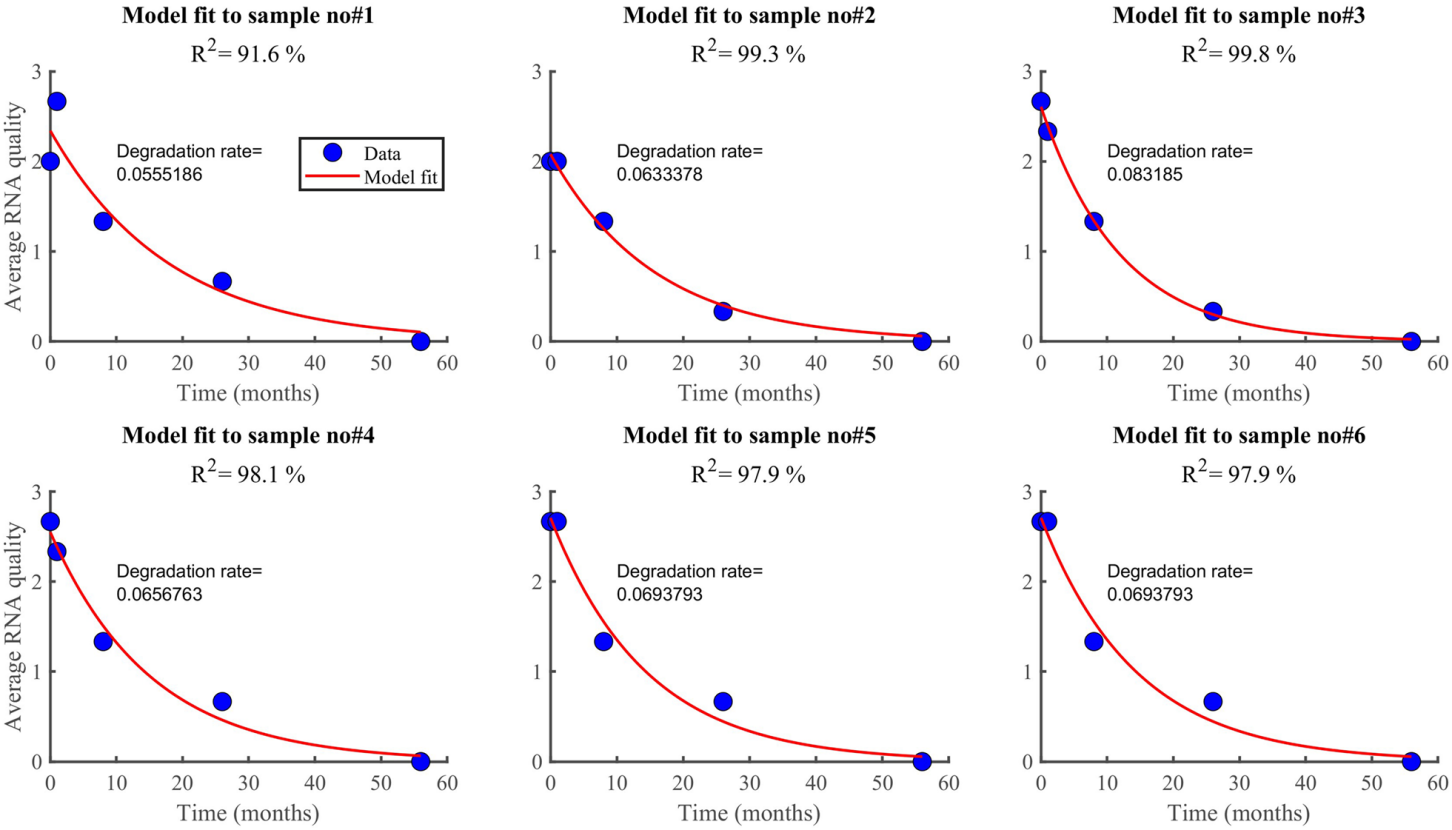
Comparison of RNA degradation rate estimated from six cassava leaf samples. The average rate of degradation is 0.0678/per month

**Fig. 9:**
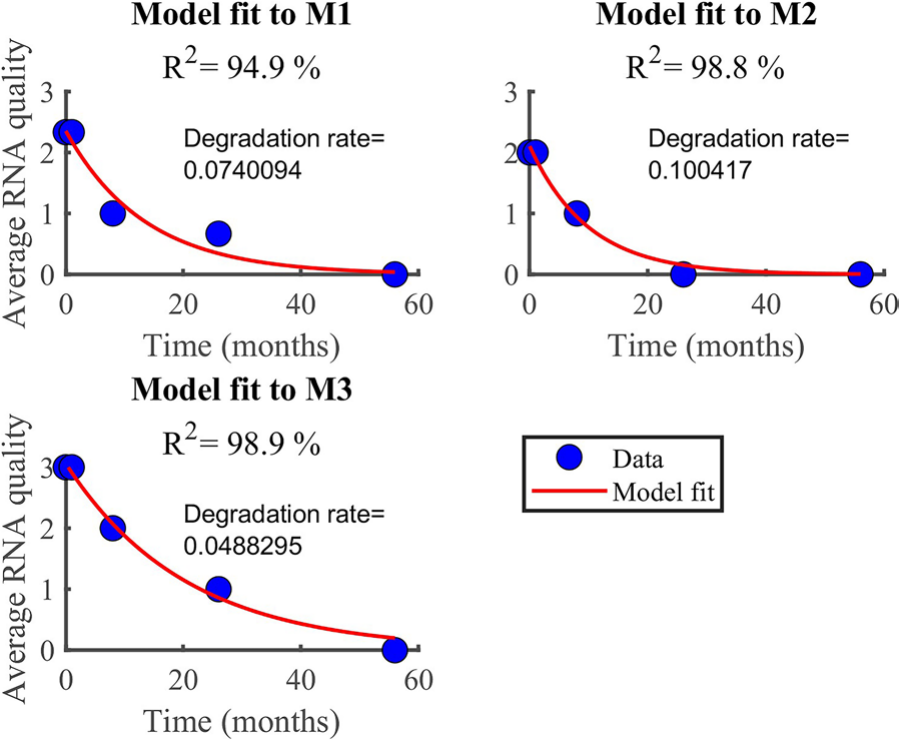
Comparison of RNA degradation rate estimated using three CTAB-based methods. The average rate of degradation is 0.0744/per month

**Fig. 10:**
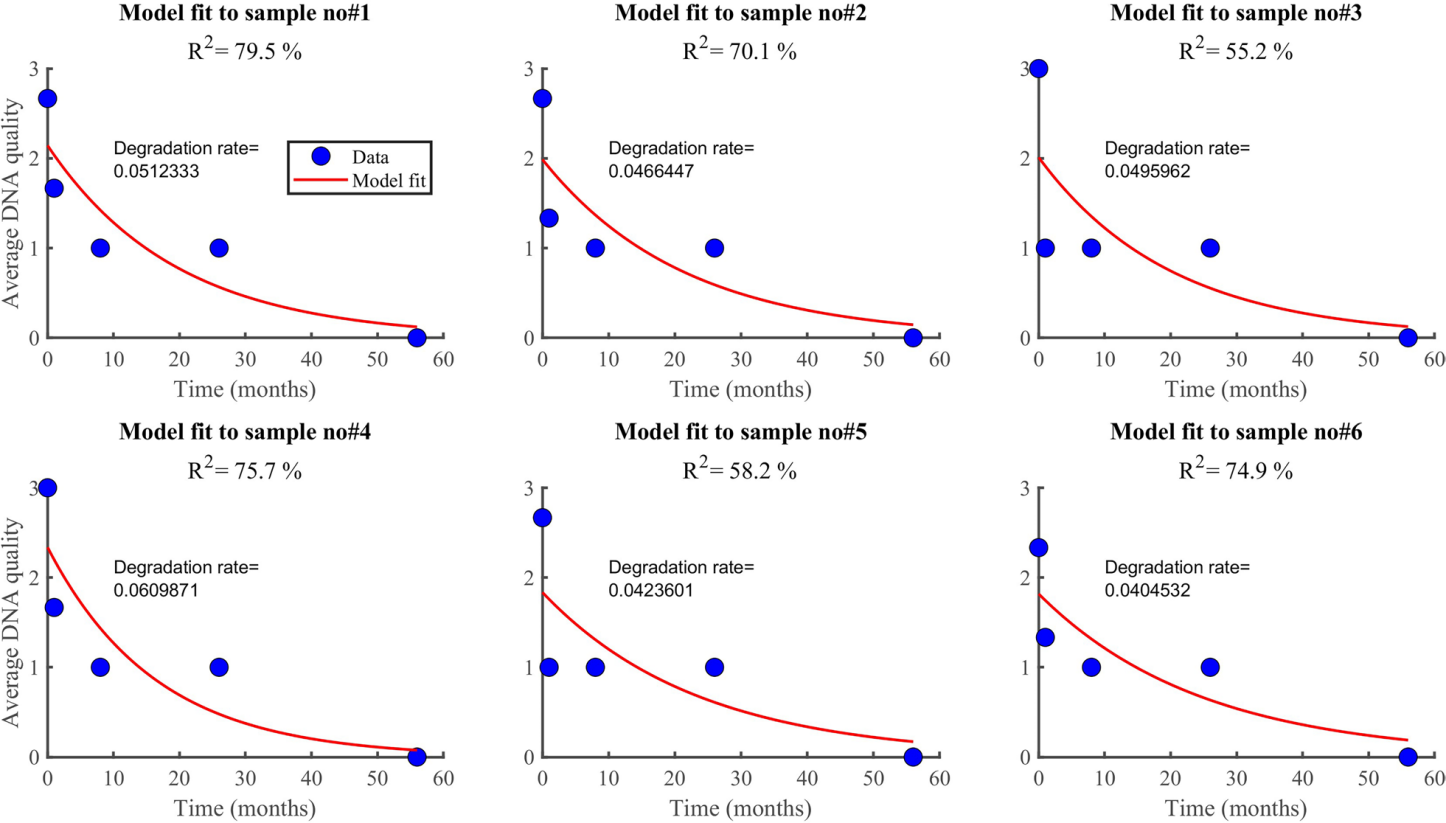
Comparison of DNA degradation rate estimated from six cassava leaf samples. The average rate of degradation is 0.0493/per month

**Fig. 11:**
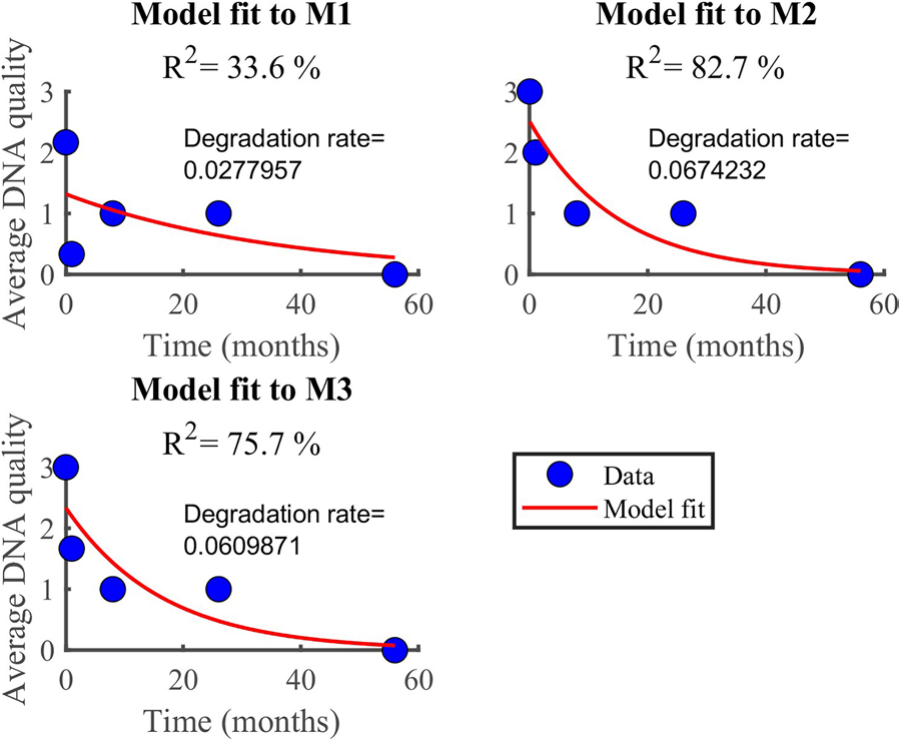
Comparison of DNA degradation rate estimated using three CTAB-based methods. The average rate of degradation is 0.0521/per month

According to the findings, RNA degrades faster (0.07/month) than DNA (0.05/month). In both datasets (Fig. 6, 7), the model (equation 2) explained more than 90% of the variability observed in the RNA data (i.e., *R*^2^ > 90%). However, the model explained only about 34% when applied to DNA quality data averaged by the method used. Generally, the model fitted well the RNA quality data than the DNA data, regardless of the method or the samples. The average rate of RNA deterioration estimated using the two datasets is 0.0678/per month and 0.0744/per month for a sample and method-based aggregation, respectively (Fig. 8, 9). The DNA degradation rates are 0.0493 and 0.0521 for sample-based and method-based aggregation, respectively (Fig. 10, 11). Overall, RNA quality degraded much faster than DNA quality (Table 6).

**Table 6:** Average degradation rate for RNA and DNA quality.

Data type	Degradation rate by sample I.D	Degradation rate by method type	Average degradation
	[month^−1^]	[month^−1^]	[month^−1^]
RNA quality	0.0678	0.0744	0.07
DNA quality	0.0493	0.0521	0.05

Data were averaged based on the number of samples and methods used

### CBSD symptoms observation and CBSVs detection

**Fig. 12:**
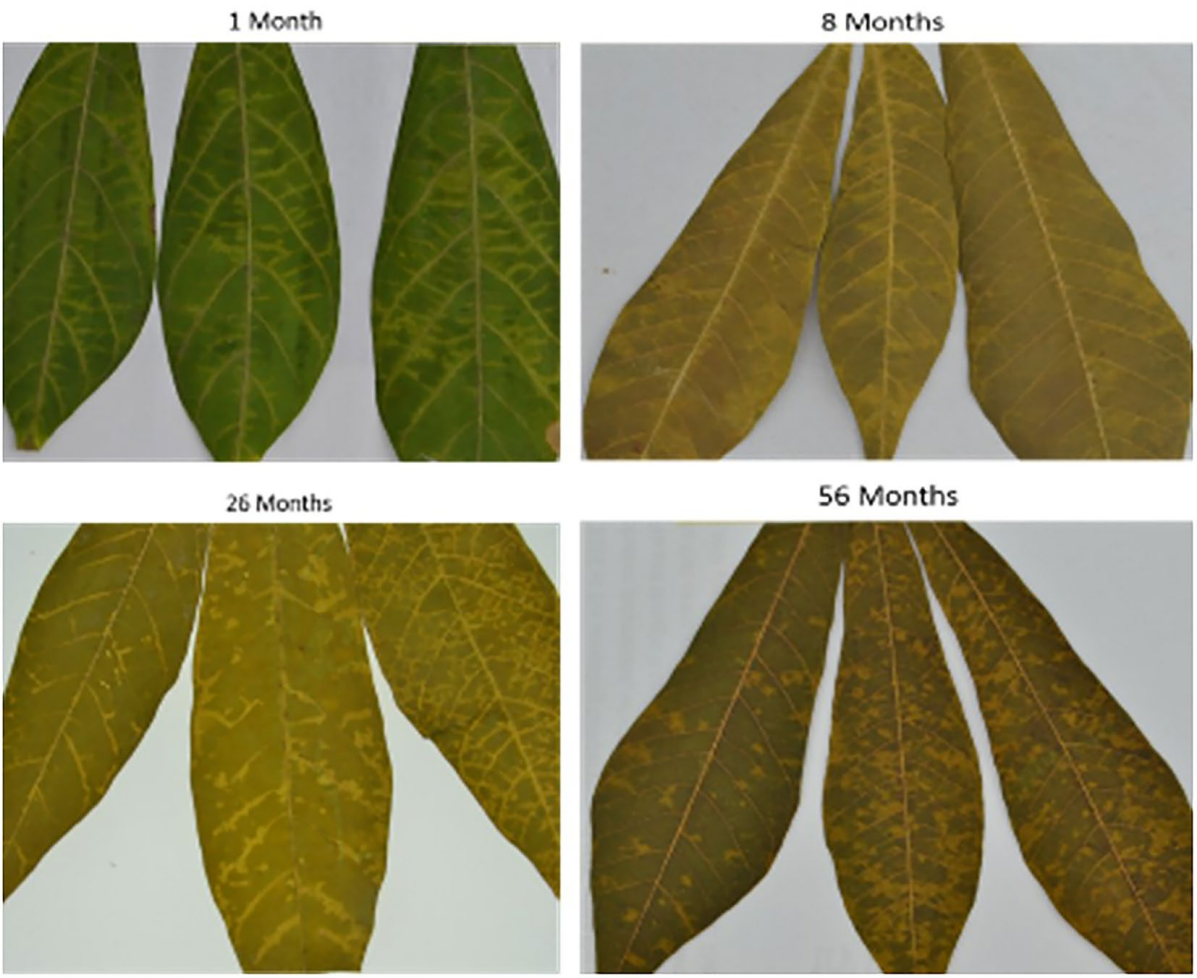
Cassava leaf samples showing varying symptoms of Cassava Brown Streak Disease (CBSD). Samples display secondary and tertiary venal chlorosis and irregular yellow blotchy

**Fig. 13:**
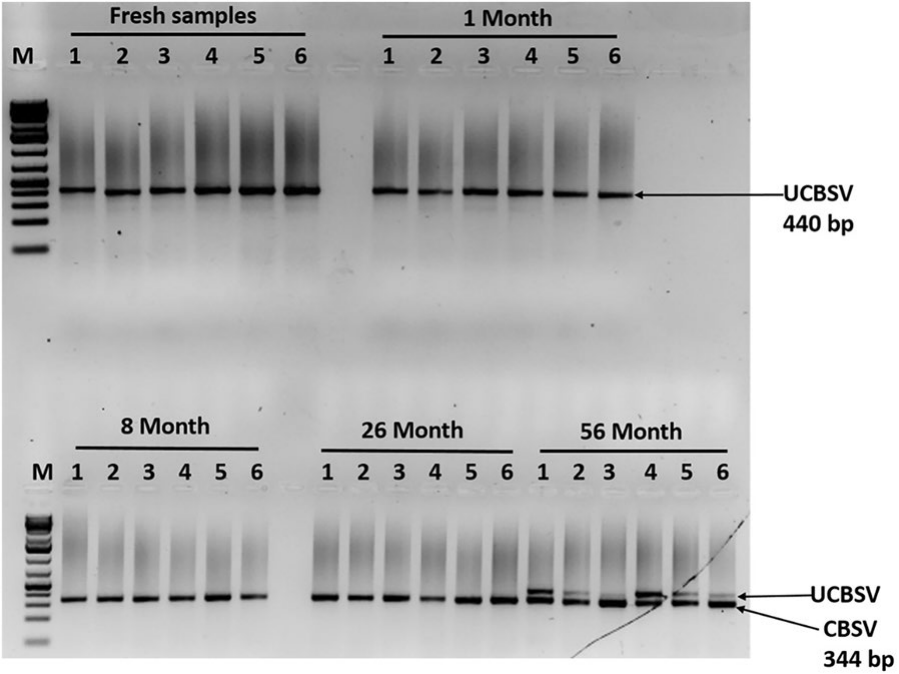
PCR results showing amplification of CBSV and UCBSV. Fresh samples and 1- month-old samples were infected with UCBSV whereas 8 and 26-month-old were infected with CBSV. The 56 month-old samples were co-infected with both CBSV and UCBSV. M is 1 kb plus DNA ladder marks 344 and 440 bp for CBSV and UCBSV respectively

**Fig. 14:**
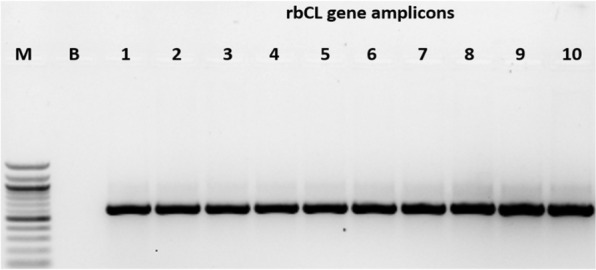
Amplification of chloroplast ribulose-bisphosphate carboxylase gene (rbcL) using P609 and P610 primer pairs. While M represents 1 kb plus DNA ladder, **B** represents buffer control respectively

CBSD symptoms were visible in fresh samples, 1-month, 8 months, 20-month, and 56-month stored samples. Chlorotic patches that eventually turned into blotches, secondary and tertiary vein clearing, and yellowing were all common CBSD symptoms (Fig. 12). In 8-month stored samples, the colour of the leaf started to change from green to purple. Still, symptoms were present when compared to 20 month and 56 month samples. Furthermore, nucleic acid obtained from M3 was used to detect CBSV and UCBSV. Fresh leaf and 1-month samples from Kimara (unknown variety) were infected with UCBSV, while 8- and 26-month samples (Mkombozi variety) from Mbinga were infected with CBSV. The optimized method recovered sufficient nucleic acids for RT-PCR from the 56-month-old Kalingisi variety collected from Mbinga District. Also, the method detected CBSV and UCBSV at expected amplicon sizes of 344 and 440 bp, respectively (Fig. 13). The chloroplast geneRibulose-1,5- Bisphosphate Carboxylase (rbcL) as barcode DNA for plant species identification was successfully amplified in all samples stored at different periods (Fig. 14).

## Discussion

### DNA and RNA quantity and quality based on storage periods

The need for intact and high-quality DNA and RNA is critical for obtaining reliable sequence results and subsequent analysis and application. Our study revealed that cassava leaves stored for a more extended period had significantly lower DNA and RNA quality than those stored for a shorter period (Additional file 1). The quality of DNA and RNA extracted from tissues stored for one day and one month was significantly higher than those obtained from tissues stored for 8, 26, and 56 months. These findings are consistent with those of [22–25], who reported that the quantity and quality of DNA and RNA in different tissue samples decreased with sample storage duration. Our results have shown the highest concentration of DNA was obtained in degraded 56-month-old samples. This information alerts us not to rely on concentration when sensitive molecular techniques such as quantitative PCR (qPCR) and gene expression microarrays are employed [26]. Nucleic acids begin to degrade quickly after the tissue is removed from the organism because when cells die, and compartments dissolve, significant amounts of nucleases, proteases, and other degradative molecules are released [27]. Enzymatic processes, hydrolytic attack, oxidation, and S-adenosyl methionine transfer of methyl are all frequent ways for nucleic acids to be degraded, and many of these processes become more common when a cell is damaged or dies. These processes also occur during sample dehydration, which is relatively fast. This could be the reason for the low-quality DNA and RNA obtained from old samples in this study. (Additional file 1).

Furthermore, in many parts of the world, temperatures regularly exceed 30 °C, accompanied by high humidity, which accelerates the rate of degradative processes for unprotected hydrated nucleic acids [28, 29]. The degradation processes must be avoided if the DNA and RNA are to be used for detailed molecular studies that require high-quality and intact N.A. Minimizing the time between collection and extraction is one of the easiest ways to ensure that high-quality nucleic acids are obtained. After sampling, nucleic acids must be isolated from any nuclease activity and other damaging reactants as soon as possible. Even though CBSV and UCBSV, as well as Chloroplast Gene-Ribulose-1,5Bisphosphate Carboxylase (rbcL) as plant species identification barcode DNA, were successfully amplified for very old samples in this study, reducing storage time is still the best option.

### DNA and RNA quantity and quality based on the extraction method

The amount and quality of DNA and RNA obtained using the existing CTAB methods (M1 and M2) were generally low compared to M3 used in this study. Our modified method recovered enough high-quality DNA and RNA than the existing two CTAB methods. M3 was developed by optimizing the existing two CTAB protocols (M1 and M2) and outperforming them to mitigate the effects of phenolic compounds and other inhibitory substances while avoiding using organic solvents such as 2-mercaptoethanol. This method, which included the addition of polyvinylpyrrolidone (PVP) (2.0% [wt/vol] final concentration) to the original protocols (M1 and M2) (Table 1) was crucial in improving the reproducibility of DNA and RNA extractions from various plants and other organisms including bacteria, fungus, marine organisms like fish and sea cucumbers. Isolation of genomic DNA and total RNA using the described M3 was quick and straightforward, taking less than 29 min.

The success of the optimized CTAB method in obtaining high-quality genomic DNA and total RNA from different examined species demonstrated the broad applicability of the method. Adding a higher concentration of PVP (2.0%) with a lower molecular weight to the extraction buffer increased the quality of the isolated DNA in this study. Previous studies [30, 31] have advocated using PVP at 2.0% (w/v) to eliminate phenolics. Antioxidants like PVP and SDS are commonly used to address phenolic and improve the colour of the obtained nucleic acid [32]. Low molecular weight PVP has a lower tendency to precipitate with nucleic acids with high molecular weight PVP, resulting in a sufficient amount of polyphenol-free DNA [33]. In this study, the purity of the extracted DNA was excellent, implying that the preparations were sufficiently free of proteins and polyphenolic/polysaccharide substances, as indicated by [34]. Reduced nucleic acid recovery and centrifuge time, as well as the number of handling processes used in this study, may have influenced the production of high-quality DNA and RNA.

### RNA and DNA degradation rate

According to the findings, RNA degrades faster (0.07/month) than DNA (0.05/month). Higher levels of DNA and RNA degradation were high for samples that had been stored longer. Our results agree with other studies that report that RNA degrades faster than DNA [21, 24]. DNA degradation is much slower than RNA degradation, and some RNAs have half-lives of minutes to hours [35]. Significant DNA degradation can be seen immediately after removing leaf tissue from the main plant, while RNA degradation can be seen seconds after sampling [36]. DNA has been found in dried tissues of numerous plant species months to millennia after the organism has died [37]. Although DNA may be detected in tissues that have been dried for a long time (up to hundreds of years in the case of fungi and tens of millennia for plants), deterioration can be seen soon after the cells begin to die [36, 38]. One important application of these rates is the estimation of half-life for RNA and DNA quality. This is the time it takes for RNA and DNA to degrade to 50% of their original quality, which is vital for plant disease planning and management purposes.

### CBSD symptoms observation and CBSVs detection

CBSD symptoms were visible in samples stored for 1 month, 8 months, 26 months, and 56 months. CBSD symptoms included chlorotic patches that eventually turned into blotches, secondary and tertiary vein clearing, and yellowing was observed. After 8 months of storage, the color of the leaf began to change from green to purple. Our findings showed that studying viral dynamics in plants necessitates a thorough understanding of plant structural and biochemical traits. According to our study, relying on visual means of virus identification is not recommended because symptom expression does not always indicate plant infection status. Brown streak-like disease symptoms are also caused by nutrient deficiency which affects the severity score during field disease surveillance. CBSV and UCBSV were detected in fresh and dried cassava leaf samples storage for different durations. Fresh samples and those stored for a short period yielded high-quality RNA suitable for RT-PCR, allowing for easy and fast detection of CBSV and UCBSV. The results are consistent with those of [39]. Studies have shown that leaf tissue samples of virus-infected plants degrade rapidly during transportation or storage, necessitating immediate appropriate sampling and preservation [40]. To store virus-infected samples preservation methods such as dry-ice, liquid nitrogen, and ultracold freezer have been employed to store virus-infected samples [41]. In this case, we propose using a herbarium as a cheaper alternative preservation mentioned above techniques. Sample collection in remote areas takes more than two weeks, necessitating affordable and proper storage methods, particularly for developing countries. RNAlater has been shown to preserve RNA and DNA in plant tissue for several weeks at room temperature and is recommended for PCR and genome sequencing analyses [42]. However, due to costs and customs issues, this is impractical when thousands of samples are collected for virus distribution and epidemiological studies. In general, we discovered that the RNA extraction reagent used significantly impacted detection rates. It should be noted that efficient and reproducible RNA extraction is critical for pathogenic virus detection and sequencing [41].

## Conclusion

The quantity and quality of DNA and RNA extracted from plant samples deteriorate with storage time, with RNA degrading faster than DNA. Fresh and 1 month-old samples had significantly higher DNA and RNA quantity and quality than samples stored for longer periods of 8, 26, and 56 months. The modified method (M3) used in this study recovered a higher quality and quantity of nucleic acids than the existing CTAB methods (M1 and M2). The degradation rate of RNA was estimated to be 0.0678/month for sample-based aggregation and 0.0744/month for method-based aggregation. In comparison, the degradation rate of DNA was estimated to be 0.0493/month for sample-based aggregation and 0.0521/month for method-based aggregation. The CBSV and UCBSV were detected using nucleic acids extracted from M3 using herbarium samples stored for up to 56 months. Chlorotic patches, secondary and tertiary vein clearing, and yellowing were common symptoms of CBSD. Additionally, the rbcL gene was successfully amplified in all samples stored at different periods, making it a reliable DNA barcode for plant viral species identification and detection.

### Supplementary Information

**Additional file 1.** Quality of DNA and RNA using method 1 and 2 (**Figure 4**) and optimized method 3 (**Figure 3**). Herbarium-stored samples displaying varying symptoms of CBSD (**Figure 11**). **Figure 12** showing PCR amplification of CBSV (344 bp) and UCBSV (440 bp).

## Data Availability

All data generated or analyzed during this study are included in this published article.
