# Supplementary material for: Assessing the effect of sample storage time on viral detection using a rapid and cost-effective CTAB-based extraction method
Source: Plant Methods. 2024 May 8;20:64. doi: 10.1186/s13007-024-01175-6 (PMC11080235; doi:10.1186/s13007-024-01175-6)
Supplement: Supplementary file 1 — Additional file 1. Quality of DNA and RNA using method 1 and 2 (Figure 4) and optimized method 3 (Figure 3). Herbarium-stored samples displaying varying symptoms of CBSD (Figure 11). Figure 12 showing PCR amplification of CBSV (344 bp) and UCBSV (440 bp). [file 13007_2024_1175_MOESM1_ESM.docx]

**Additional file materials**

**Additional file figures**

This supplementary material presents the original uncropped Figure 3, Figure 4, Figure 11 and Figure 12 in the main text.

**
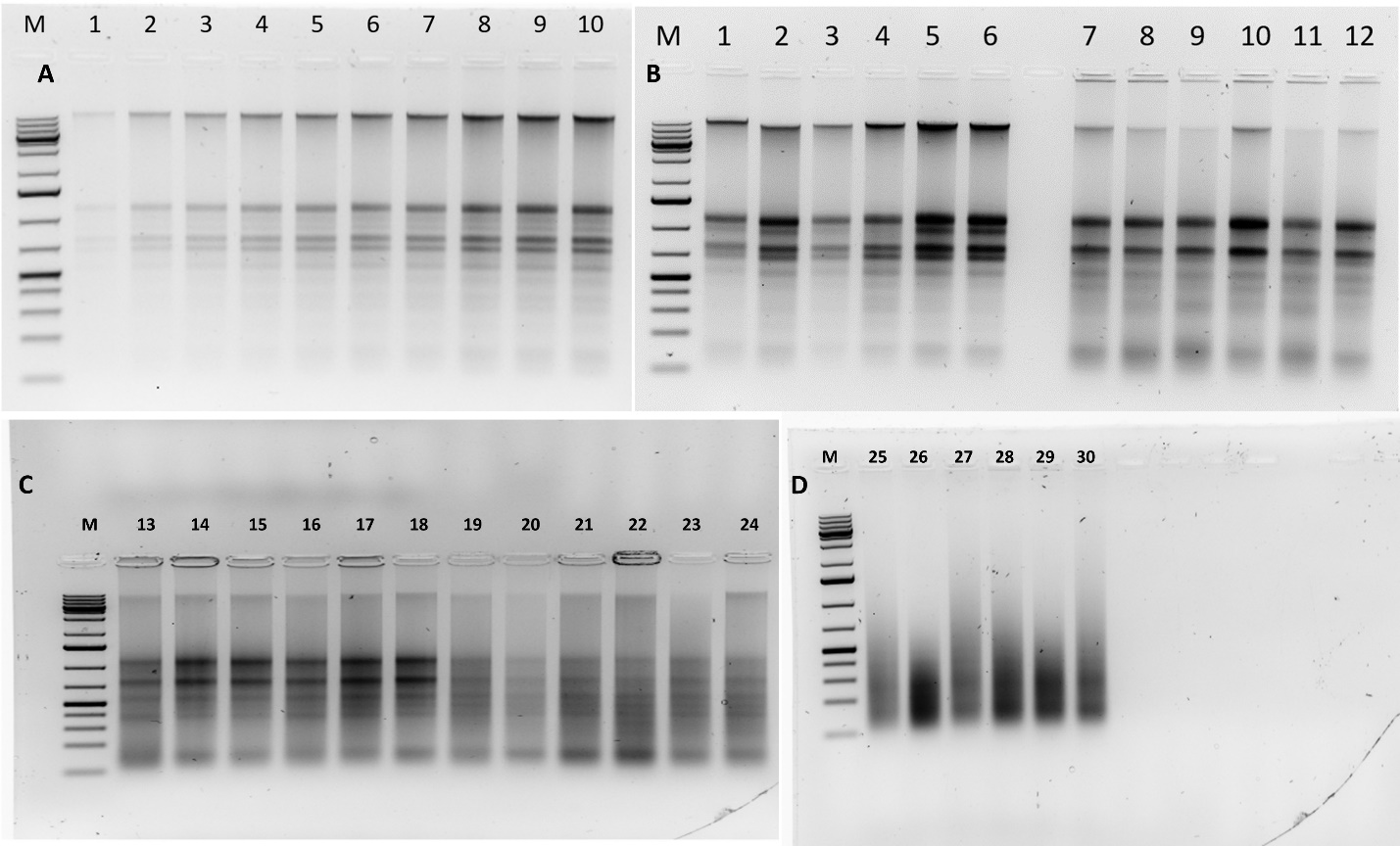
**

**Figure 3.** Quality of DNA and RNA isolated using optimized method 3 (M3). **A)** 1 to 10 are total nucleic acids extracted from a fresh cassava leaf sample using our optimized method then diluted to 200ng to 2000ng. 1 (200ng), 2 (400ng), 3 (600ng) 4 (800ng), 5 (1000ng), 6 (1200ng), 7 (1400ng), 8 (1600ng), 9 (1800ng) and 10 (2000ng). Following experiments, 1800 ng was used as the standard in subsequent gels, **B)** Samples 1 to 6 are genomic DNA and total RNA isolated from fresh leaf samples and 7 to 12 are herbarium 1-month old using a modified method. 800 ng of total nucleic acids were used in the agarose gel, **C)** Samples 13-18 and 19-24 are total nucleic acids (RNA & DNA) isolated from 8 and 26 month-old herbarium respectively using modified M3, **D)** Total nucleic acids isolated from 56 month-old herbarium samples (samples 25-30) using a modified (M3) method.


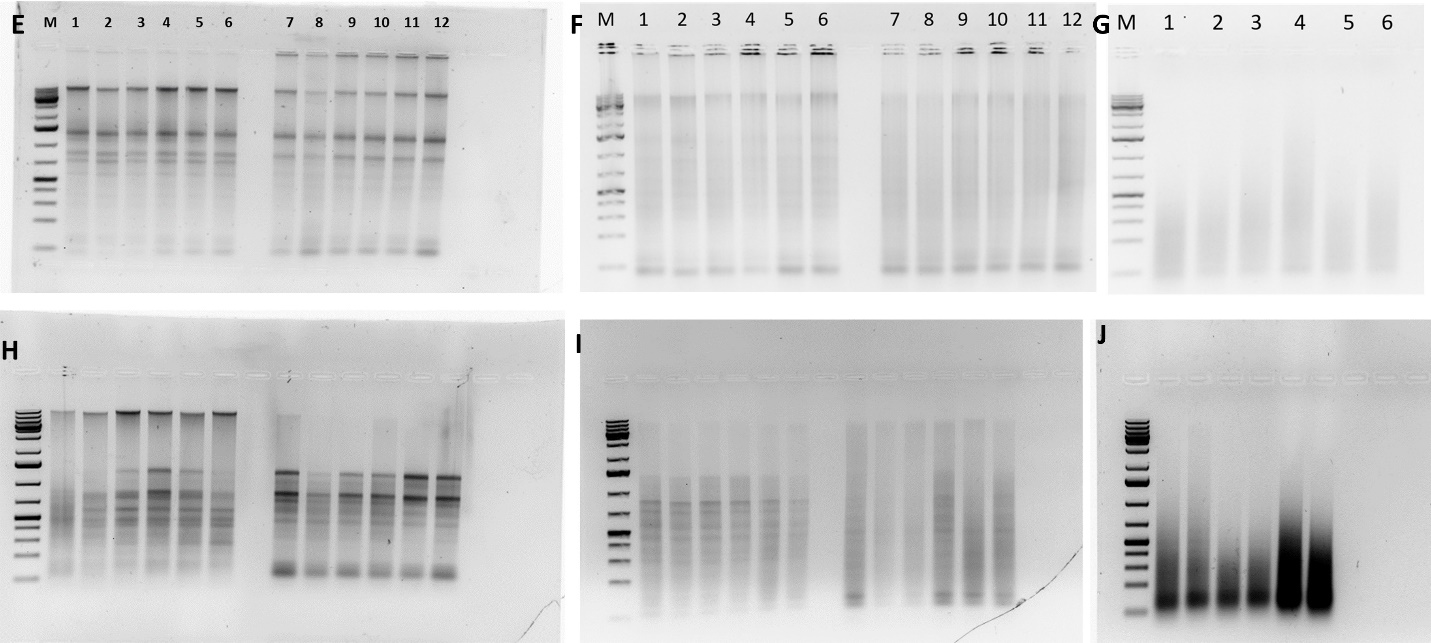


**Figure 4.** Total nucleic acids isolated using extraction methods 1 and 2, **E**) DNA and RNA isolated from fresh cassava samples 1-6, and 1-month-old herbarium samples 7-12 isolated using method 2, **F**) Samples 1-6 and 7-12 are nucleic acids of 8 and 26-month-old herbarium samples respectively isolated using method 2. **G**)56-old samples showing degraded RNA and DNA isolated using method 2, **H**) DNA and RNA extracted using method 1. Samples 1-6 are fresh cassava leaf samples and 7-12 are 1 month old, **I**) Total nucleic acid isolated using method 1. Samples 1-6 are 8 months old while 7-12 are 26 month old samples and J) Total nucleic acids isolated from 56 months’ old herbarium cassava leaf samples (samples 1-6) using method 1.


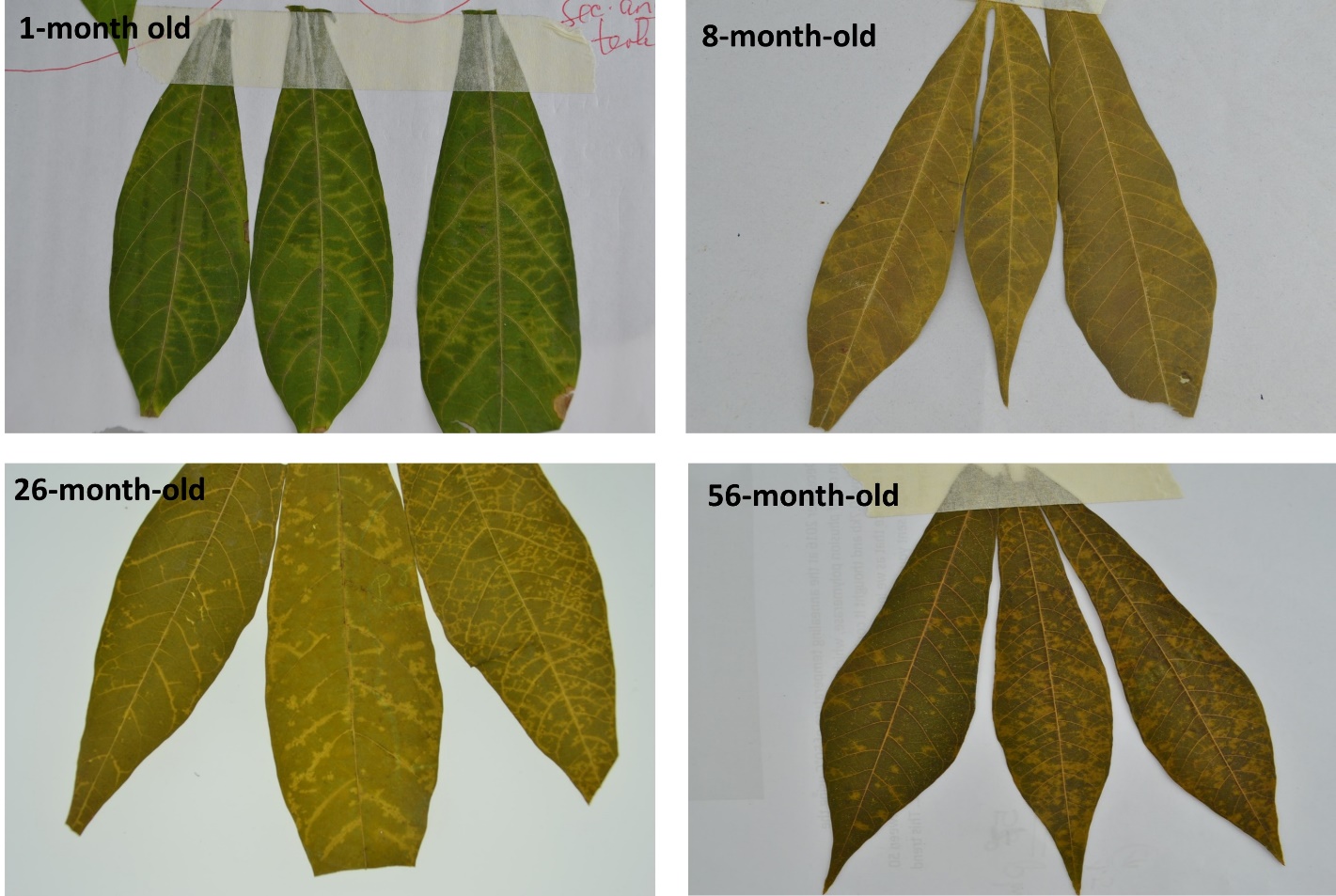


**Figure 11**. One-month old herbarium cassava leaf samples displaying cassava brown streak symptoms (vein clearing), Eight month-old cassava leaf samples, 26-month old herbarium cassava leaf samples and 56 month-old herbarium samples displaying cassava brown streak symptoms (chlorotic spots).


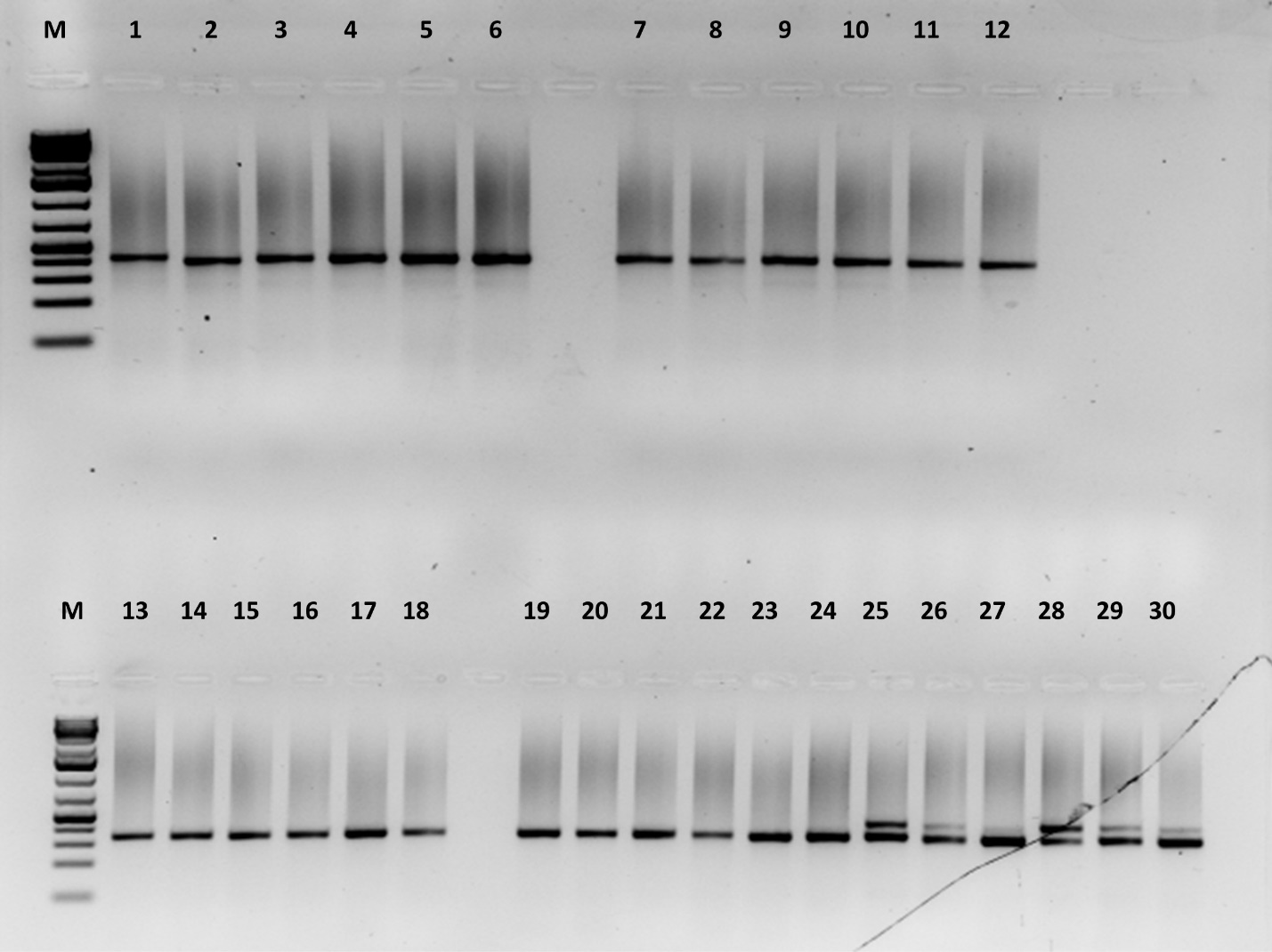


**Figure 12.** PCR results showing amplification of CBSV and UCBSV. Fresh samples and 1-month-old samples (1-6 and 7-12) were infected with UCBSV whereas 8 and 26-month-old (13-18 and 19-24) were infected with CBSV. The 56-month-old samples (25-30) were co-infected with both CBSV and UCBSV. M is 1kb plus DNA ladder marks 344 and 440 bp for CBSV and UCBSV respectively.
